# The Lively Flea: A Wonderfully Constructed and Pugnacious Little Pest1From the New York World.

**Published:** 1896-08

**Authors:** 


### THE LIVELY FLEA: A WONDERFULLY CONSTRUCTED AND PUGNACIOUS LITTLE PEST TO HUMANITY.[1]

THE flea is an insect peculiarly difficult to combat, on account of its strength and agility and the fact that it readily transfers itself from the person to an inanimate object. It is less of a reproach to be troubled by fleas than by any class of parasites. These insects are found in all walks of life and do not hesitate to attach themselves to ladies of the highest social position.

Nearly every family of animals seems to have its own particular flea, which does not trouble any other family. The furry animals are much more frequented by fleas than man, and fortunately these fleas seldom flourish upon the human frame.

Cats and dogs are always well provided with fleas, and so are moles, squirrels, mice, rats, and rabbits. These fleas have lesser fleas to prey upon and bite them, and so proceed *ad infinitum*, as Dean Swift has remarked.

The human flea when studied scientifically is truly a beautiful and interesting object. In shape it is flattened from side to side and has a high-ridged back. The body is covered with a hard, red, slippery skin, rendering it extremely difficult to hold between the fingers and crush.

It has three pairs of legs, which increase in length from before backward, giving the insect somewhat the aspect of a kangaroo. The first joint of a flea's leg, that nearest the body, is enormously large, and enables it to perform the leaping feats for which it is famous. The legs terminate in long, curved claws, of great use in aiding the insect to work its way through the garments of its host. They are covered with bristly brown hairs.

The flea's mouth is one of its most admirable features. It is of the type known as suctorial, being adapted for the swallowing of liquid food obtained by a process of perforation. It consists of one single organ and four pairs of organs. The upper lip is a saw-edged bristle, perforated by a minute canal. The mandibles pointed downward on each side of it are two straight blades, provided with six hundred sharp little teeth. The maxillæ are two sharp-pointed triangular pieces, with a knife-like edge on one side.

[1] From the New York World.

The human flea is a very pugnacious animal, and captured specimens can be made to give a pretty pugilistic exhibition. A scientist speaks of two females whom he had put in a glass tube where they were to deposit eggs, as "becoming rampant, confronting one another like microscopic kangaroos."

The flea has the power of emptying the contents of its stomach whenever an opportunity of obtaining a better and a fresher supply of blood presents itself.

Unoccupied rooms will sometimes be found to contain many fleas, showing that they can subsist for a long time without human blood. They live in hope.

The human flea is an epicure. It gives little trouble to persons with tough, dried-up skins, and shows great judgment in selecting young women and children with nice, tender skins.

The flea's eggs are laid not upon the human body, where it spends most of its life, but upon rugs and mats and in dark places where there is plenty of dust and dirt. It is therefore a matter of ordinary cleanliness to keep the insects in check. Why there should be great epidemics of fleas in some years is a mystery.

On to Buffalo!

